# The Antigastric Cancer Effect of Triptolide is Associated With H19/NF-κB/FLIP Axis

**DOI:** 10.3389/fphar.2022.918588

**Published:** 2022-08-30

**Authors:** Weiwei Yuan, Jinxi Huang, Shanshan Hou, Huahua Li, Liangyu Bie, Beibei Chen, Gaofeng Li, Yang Zhou, Xiaobing Chen

**Affiliations:** ^1^ Department of General Surgery, The Affiliated Cancer Hospital of Zhengzhou University & Henan Cancer Hospital, Zhengzhou, China; ^2^ Department of Pharmacy, Zhejiang Pharmaceutical College, Ningbo, China; ^3^ Department of Medical Oncology, The Affiliated Cancer Hospital of Zhengzhou University and Henan Cancer Hospital, Zhengzhou University, Zhengzhou, China; ^4^ Children's Hospital Affiliated to Zhengzhou University, Henan Children's Hospital, Zhengzhou Children's Hospital, Zhengzhou University, Zhengzhou, China

**Keywords:** triptolide, TNF-*α*, gastric cancer, H19, NF-κB

## Abstract

**Background and Objective:** Triptolide (TP), one of the fat-soluble components extracted from the Chinese medicinal herb *Tripterygium wilfordii* Hook F. (TWHF), possesses strong antitumor bioactivities, but its dose-dependent side effects restrict its wide application. This study was designed to investigate whether inflammatory factors increased the antitumor effects of the nontoxic dose of TP on gastric cancer cells and tried to explore the possible molecular mechanisms.

**Method:** AGS and MKN45 cells were treated with different doses of TP and TNF-*α*. Cell viability and apoptosis were detected *in vitro*. In addition, NF-κB mediated prosurvival signals and cytoprotective proteins, especially FLICE-inhibitory protein (FLIP), were detected to determine their effects on TP/TNF-*α*–induced apoptosis. Moreover, the function of lncRNA H19/miR-204-5p/NF-κB/FLIP axis was investigated *in vitro*, and the antigastric cancer effect of TP plus TNF-*α* was proved in the mice xenograft model.

**Result:** *In vitro* experimental results showed that TP pretreatment promoted apoptosis in AGS and MKN45 cells upon TNF-*α* exposure. TP/TNF-*α*–mediated apoptosis was partly mediated by the inhibitory effect of NF-κB–mediated FLIP expression. Oncogene H19 lying in the upstream pathway of NF-κB played a vital role upon TNF-*α* exposure, and bioinformatics analysis proved that H19 participated in TP/TNF-*α*–induced apoptosis *via* binding of miR-204-5p. Lastly, a low dose of TP and TNF-*α* inhibited the tumor weight and tumor volume of AGS and MKN45 cells *in vivo*.

**Conclusion:** TP pretreatment increased apoptosis in TNF-*α*–stimulated gastric cancer cells, which are dependent on the disruption of the H19/miR-204-5p/NF-κB/FLIP axis. Cotreatment of TP and TNF-*α* is a better option for enhancing the anticancer effect and lowering the side effect of TP.

## Introduction

The development of cancer and cancer therapy response is tightly regulated by inflammation, which facilitates tumor progression upon continuous inflammatory stimulation and accelerates cancer recovery by provoking immune response (Zhao et al., 2021). Inflammatory stimulation could also strengthen the efficacy of antitumor drugs. For example, 5-fluorouracil enhances the chemosensitivity of gastric cancer to TRAIL, and Tanshinone IIA sensitizes TRAIL–induced apoptosis in glioblastoma through death receptor (Zhou X. et al., 2021; Li et al., 2021). Natural products extracted from plants, animals, or microbes play an essential role in the human struggle against diseases for centuries (Rao et al., 2019; Wang et al., 2020). It is reported that triptolide (TP), an active ingredient of a Chinese herbal plant *Tripterygium wilfordii* Hook F (TWHF), has antimicrobial, immunomodulatory, antitumor, antirheumatic, and antiinflammatory activities associated with serious systemic toxicity (Tong et al., 2021). Although TP possesses strong antitumor bioactivity, the dose-dependent side effects limit its application. Exploring a better solution for enhancing the antitumor bioactivity and lowering the side effects of TP has great significance. Previously research found that TP pretreatment increased the sensitivity of hepatocytes upon TNF-*α*/LPS stimulation (Yuan et al., 2019). The mechanistic study revealed that TP/TNF-*α* (TP/LPS) induced apoptosis in hepatocytes *via* downregulating NF-κB–mediated cellular FLICE-inhibitory protein (FLIP) expression (Yuan et al., 2019; Yuan Z. et al., 2020). However, whether we can take advantage of this feature of TP to increase its antitumor activity remains elusive.

Gastric cancer is ranked second among other cancers in terms of incidence and mortality in the population of China. Statistical data show that the incidence of stomach cancer is 679.1 per 0.1 million and mortality is 498 per 0.1 million. Late-stage diagnosis along with poor prognosis is the main cause of the high mortality rate in gastric cancer patients. Therefore, understanding the biological mechanisms and seeking a better treatment plan is the need of the hour. Previous antitumor studies of TP on gastric carcinoma mainly focused on the direct cytotoxicity of TP, while a high dose of TP might also affect the physiological function of normal cells (Jiang et al., 2001; Chang et al., 2007; Wang et al., 2014). Thus, a scientific approach for improving the antitumor effects of TP along with lessening its side effects is desired.

When TNF-*α* binds to TNF-R1, it leads to the activation of NF-κB with a subsequent increase in the protein expression of FLIP. This cellular FLICE-like inhibitory protein has the main role in regulating cell fate. After the cytoplasmic transfer of the TNF receptor–associated death domain, receptor-interacting protein kinase 1 (RIPK1), and TNF receptor-associated factor 2 (TRAF2) through their death domains, coupled with Fas-associated death domain (FADD) and Caspase-8, create a complex named complex-IIa (Micheau and Tschopp, 2003). When NF-κB synthesizes FLIP, the formation of Caspase-8/FLIP dimer arrests the activity of Caspase-8. However, the inactivation of NF-κB leads to the activation of Caspase-8, which can cause cell death through the apoptosis pathway upon TNF-*α* stimulation. Previous studies revealed that TP/TNF-*α* (TP/LPS) induced apoptosis in hepatocytes *via* downregulating NF-κB–mediated FLIP expression (Yuan et al., 2019). However, the mechanism behind TP–induced NF-κB inhibition is still unknown, and whether TNF-*α* can enhance the antitumor effect of TP remains unclear.

Among 75% of transcribed RNA molecules, long noncoding RNA (lncRNA) is supposed to be involved in various biomolecular processes, such as transcriptional modulation, translational regulation, and epigenetic mechanism. Research studies have discovered that the expression of lncRNAs, including MALAT1, H19, MEG3, and TUSC7, markedly control gastric cancer cell migration, proliferation, invasion, metastasis, cell cycle, tumorigenicity, and apoptosis (Huang and Yu, 2015; Yang et al., 2015; Deng et al., 2016; Xie et al., 2016). In addition, targeting miRNA for binding to mRNA following protein regulation is an important process for lncRNAs to exert their physiological functions (Chen et al., 2021). It is widely accepted that some lncRNAs regulate the activity of NF-κB and ultimately participate in inflammation and immune response (Gupta et al., 2020). H19 is one of the most studied lncRNAs in carcinogenesis that plays a key role in the multistep process of carcinogenesis including genomic irregularity, translational fluctuation, unstable proliferation, stress management, and metastasis (Raveh et al., 2015a). It is widely accepted that the function of H19 can be divided into two parts: the reservoir of miR-675 to suppress its target genes and the binding to miRNAs or proteins to modulate their function (Raveh et al., 2015b). The previous study showed that TNF-*α* treatment also increased the level of H19, which in turn led to the activation of the NF-κB pathway by stimulating the phosphorylation of TAK1, which implied that H19 might be involved in TP/TNF-*α*–induced apoptosis (Yang et al., 2020b).

In this study, we explored whether TNF-*α* enhanced the antitumor effect of TP against gastric carcinoma. Moreover, we screened lncRNAs related to the pathogenesis of gastric cancer and tried to investigate the effect of the H19/miR-204-5p/NF-κB axis in TP/TNF-*α*–induced apoptosis in gastric carcinoma.

## Materials and Methods

### Materials

TP (CAS number 38748-32-2, purity > 98%) was supplied by SelleckChem (Houston, TX, United States). Human recombinant TNF-*α* (300-01A) and Cell Counting Kit-8 (CCK-8, HY-K0301) were obtained from Peprotech Inc. (Rocky Hill, NJ, United States) and MedChemExpress (Madison, WI, United States), respectively. Trizol reagent (R401-01), SYBR Green Master Mix (Q111-03), and Reverse Transcription Kit (R312-02) were obtained from Vazyme Biotech Co., Ltd. (Nanjing, Jiangsu, China).

Antibodies against FLIP (56343), X-linked inhibitor of apoptosis protein (XIAP, 2042), cleaved caspase-8 (9746), and cleaved caspase-3 (9661) were purchased from Cell Signaling Technology (Boston, MA, United States). Antibody against NF-B p65 (ab16502) was purchased from Abcam (Cambridge,United Kingdom). Antibodies against cellular inhibitor of apoptosis protein 1 (CIAP1) and cellular inhibitor of apoptosis protein 2 (CIAP2) (10022-1-AP), Lamin B1 (12987-1-AP), and *β*-Actin (HRP-60008) were purchased from Proteintech (Chicago, IL, United States).

### Animal and Pharmacological Treatments

All experimental procedures involving mice complied with the ARRIVE guidelines and were permitted by the Animal Ethics Committee, Zhengzhou University. To explore the antitumor effects of TP, six-week-old female athymic BALB/c nude mice were purchased from SPF (Beijing) Biotechnology Co., Ltd. and maintained under pathogen-free conditions. Nude mice were subcutaneously injected into the inguinal region with 5 × 10^6^ AGS and MKN45 cells (6 mice per group). When tumor volume reached 30–50 mm^3^, the mice were administered with TP intragastrically (125 μg/kg or 250 μg/kg) or 0.5% CMC–Na at the volume of 10 ml/kg once a day. Tumor volumes were measured every 3 days, and all the mice were sacrificed 3 weeks after TP treatment. Tumors were collected for further detection.

To investigate the antitumor effects of TP and TNF-*α*, nude mice were subcutaneously injected into the inguinal region with 1 × 10^7^ AGS and MKN45 cells (6 mice per group). The mice were administrated with TP (125 μg/kg) and TNF-*α* (5 μg/kg). TP was administrated once a day, and an intratumoral injection of TNF-*α* was administered twice a week. Mice used in this experiment were sacrificed 3 weeks after TP administration.

### Cell Culture and Cell Viability Assay

Cell lines used in this experiment, including AGS, MKN45, and HEK293T cells, were purchased from China Cell Culture Center (Shanghai, China). Cells were cultured in Dulbecco Modified Eagle’s medium (DMEM) with 10% fetal bovine serum (FBS) provided by Gibco (Grand Island, NY, United States) and incubated at 37°C in a humidified atmosphere with 5% CO_2_. TP was added 2 h before the addition of human recombinant TNF-*α* in all *in vitro* experiments.

Cell viability was detected with the CCK-8 assay in the 96-well plates. In brief, exponentially growing cells were plated at the density of 5 × 10^3^ in each well. Fresh DMEM without FBS containing different concentrations of TP as well as human recombinant TNF-*α* was added to replace the old medium 24 h after seeding. CCK-8 reagents were added at the indicated time, and cell viability was detected with Varioskan LUX (Thermo Fisher Scientific, Waltham, MA, United States).

### Cell Transfection

Cell transfection was carried out according to our published research (Zhou Y. et al., 2021). Briefly, for gene silencing of miR-204-5p or overexpression of human FLIP and H19, miRNA inhibitor or plasmid carrying the corresponding sequence were purchased from Genepharma (Shanghai, China). H19 siRNA (SR319206) and its negative control (SR30004) were purchased from Origene Technologies (Beijing, China). miRNA inhibitor or plasmid were transfected into the cells using Lipofectamine 3000 (Invitrogen, Carlsbad, CA, United States) according to the manufacturer’s protocol. AGS and MKN45 cells cultured in 12-well plates were transfected with scrambled control sequence, miR-204-5p inhibitor, H19 siRNA, pLVX-EF1*α*-IRES-puro vector, or pEX-1 vector carrying H19 sequence. Fresh DMEM medium was added to the plates to replace the transfection medium after a 24-h transfection. Next, TP (25 nmol/L) and TNF-*α* (5 ng/ml) were added to the medium 48 h after transfection. Then, cell supernatant was collected at the indicated time after TNF-*α* application. The sequences of miR-204-5p, H19 siRNA, and their negative controls are presented in Table.1.

**TABLE 1:** Sequence of target miR-204-5p.

Gene	Gene sequence (5′-3′)
NC inhibitor	CAG​UAC​UUU​UGU​GUA​GUA​CAA
miR-204-5p inhibitor	AGG​CAU​AGG​AUG​ACA​AAG​GGA​A

### Dual-Luciferase Reporter Assay

Dual-luciferase reporter assay was conducted according to the published research (Gao et al., 2019). In brief, to confirm that miR-204-5p was the direct target of H19, the sequence of wild-type H19 and the corresponding H19 mutants were inserted into psiCHECK-2 plasmid obtained from Genepharma. HEK293T cells were co-transfected with either psiCHEKC-2-H19 wt or psiCHEKC-2-H19 mut together with miR-204-5p inhibitor or negative control (NC) using Lipofectamine 3000. Dual-luciferase reporter assay was conducted according to the Dual-luciferase Reporter Assay System Kit (E1960) obtained from Promega (Madison, WI, United States).

### RNA Extraction and qPCR

Cellular RNA was isolated using Trizol reagent, and complementary DNA was synthesized from 1-μg RNA for each sample with Reverse Transcription Kit after the quantification of RNA concentration with Nanodrop 2000 (Thermo Fisher Scientific), according to the manufacturer’s instructions. The experiment was conducted on Applied Biosystems 7500 Real-Time PCR Systems (Thermo Fisher Scientific) using SYBR Green Master Mix and normalized with U6 for the detection of miR-204-5p and *β*-actin for the detection of H19. The relative mRNA expression was calculated using the ΔΔCT method, and the primers used for qPCR are listed in Table 2.

**TABLE 2:** Primer sequences used for qPCR assay in mice.

Gene	Forward primer (5′-3′)	Reverse primer (5′-3′)
*β-actin*	CCA​TGT​ACG​TTG​CTA​TCC​AG	CTT​CAT​GAG​GTA​GTC​AGT​CAG
*LncRN H19*	ACC​ACT​GCA​CTA​CCT​GAC​TC	CCG​CAG​GGG​GTG​GCC​ATG​AA
*U6*	GCT​TCG​GCA​GCA​CAT​ATA​CTA​AAA​T	CGC​TTC​ACG​AAT​TTG​CGT​GTC​AT
*miR-204-5p*	CCTTTGTCATCCTATGCC	GAA​CAT​GTC​TGC​GTA​TCT​C
*TNFAIP3*	CTC​AAC​TGG​TGT​CGA​GAA​GTC​C	TTC​CTT​GAG​CGT​GCT​GAA​CAG​C
*NFKBIA*	TCC​ACT​CCA​TCC​TGA​AGG​CTA​C	CAA​GGA​CAC​CAA​AAG​CTC​CAC​G

### Western Blot Analysis

Total cellular protein was extracted from cell lines or tumor tissues with radio-immunoprecipitation assay lysis buffer (P0013C) purchased from Beyotime Biotechnology (Shanghai, China). Nuclear proteins were separated with Nuclear Plasma Separation Kit (P0028, Beyotime Biotechnology). The concentration of protein was quantified using a BCA Kit (P0012, Beyotime Biotechnology). The cell lysate was then mixed with 5× loading buffer (P0015, Beyotime Biotechnology) and separated using SDS-PAGE with gels ranging from 8% to 12%. After transferring the proteins onto poly(vinylidene fluoride) membranes (Millipore, Danvers, MA, United States) and blocking with 5% BSA at room temperature for 1 h, the membranes were incubated with primary antibodies overnight at 4°C. The membranes were then incubated with secondary antibodies for 1 h and visualized in Tanon 5200 Chemiluminescent Imaging System (Shanghai, China) using an ECL Detection Kit (Millipore). Relative protein expression was normalized with Lamin B1 for the detection of NF-κB p65 or *β*-actin for the detection of the whole protein lysate and analyzed with Image J 1.52a (NIH, Bethesda, MD, United States).

### NF-κB Transcription Factor Activation Assay

Cell lysates were collected at the indicated time and then extracted and quantified with the kit purchased from Beyotime Biotechnology, as described above. NF-κB transcription factor activation was detected using the kit from Abcam (ab176648), according to the manufacturer’s instructions.

### Statistical Analysis

Data were analyzed using GraphPad Prism 8.01 (GraphPad Software, San Diego, CA, United States) and presented in the form of mean ± SEM. One-way analysis of variance (ANOVA) and two-way ANOVA followed by Tukey’s multiple comparison test was performed to analyze the differences between groups. *p*-values < 0.05 were considered to be statistically significant.

## Results

### TP Pretreatment Increased the Sensitivity of AGS and MKN45 Cells to TNF-*α*

**FIGURE 1:**
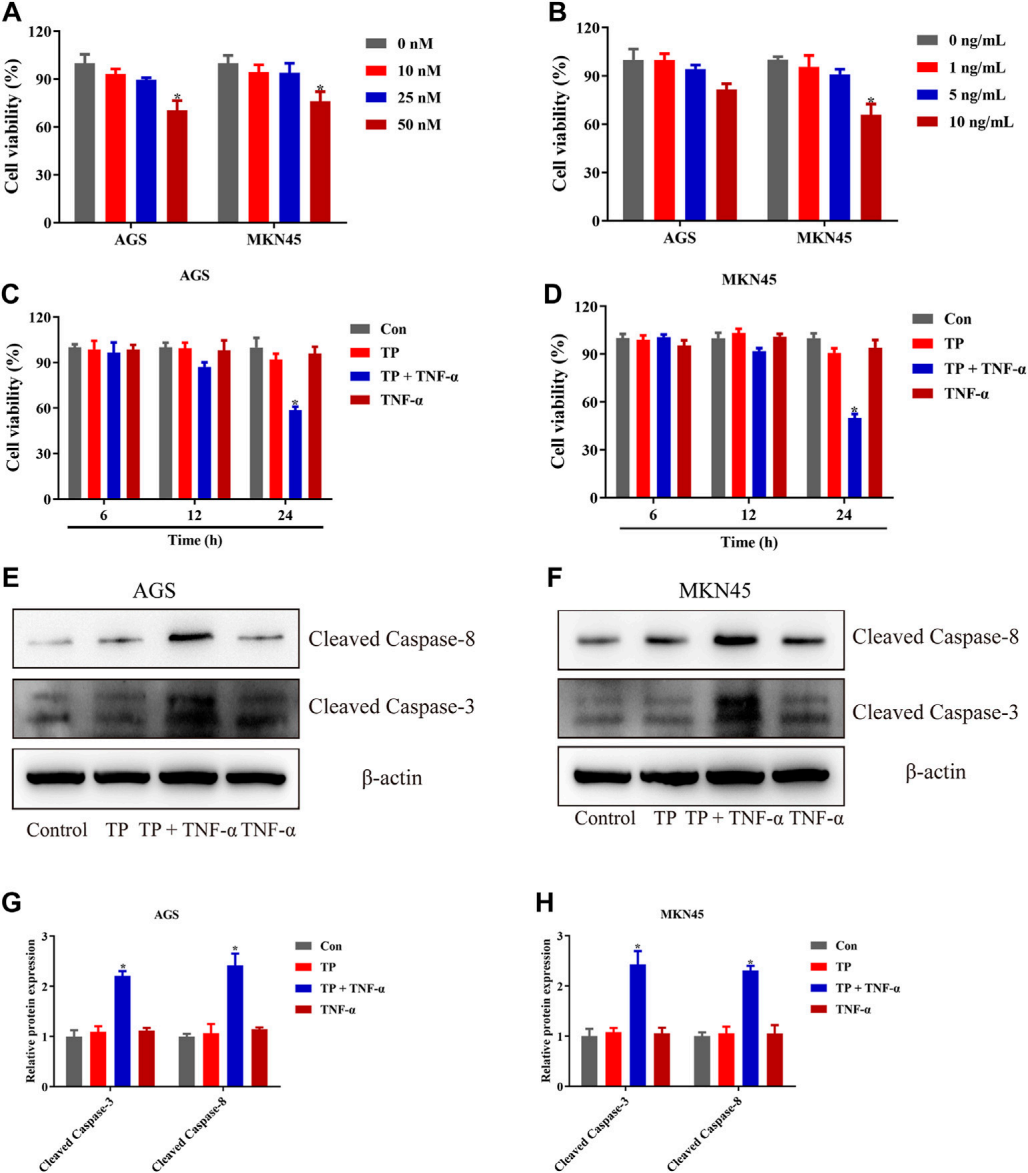
TP pretreatment increased the sensitivity of AGS and MKN45 cells to TNF-*α*. **(A)** Cell viability of AGS and MKN45 cells treated with different concentrations of TP between 10 and 50 nM for 24 h. **(B)** Cell viability of AGS and MKN45 cells treated with different concentrations of TNF-*α* ranging from 1 ng/ml to 10 ng/ml for 24 h. **(C–D)** Cell viability of AGS and MKN45 cells treated with TP (25 nM) and TNF-*α* (5 ng/ml) for 6, 12, and 24 h. **(E–H)** Representative Western blots and relative intensity of protein bands of cleaved caspase-3 and cleaved caspase-8 of AGS and MKN45 cells treated with TP (25 nM) and TNF-*α* (5 ng/ml), 24 h after TNF-*α* application, with *β*-actin as the loading control. Results were expressed as mean ± SEM, and statistical analysis was performed using one-way ANOVA or two-way ANOVA followed by Tukey’s multiple comparison test. ^*^ *p* < 0.05 (*n* ≥ 3). ^*^ *p* compared with the control group.

TP has been recognized as an antitumor active ingredient for various cancers (Noel et al., 2019). The result in Figure 1A showed that TP decreased the cell viability of two gastric cancer cell lines, AGS and MKN45, in a concentration-dependent manner. However, low doses of TP (10 and 25 nM) had little effect on cell viability. However, 50-nM concentration significantly decreased the cell viability in both cell lines. In addition, researchers reported that a high dose of TNF-*α* induces cell death, while a low dose of TNF-*α* induces cell survival instead of cell death (Brenner et al., 2015; Annibaldi and Meier, 2018). The result of Figure 1B showed that only a 10-ng/ml concentration of TNF-*α* had remarkably decreased the cell viability of MKN45 cells, which was consistent with the published articles (Oberst et al., 2011; Suda et al., 2016). Next, we treated both the cell lines with nontoxic concentrations of TP (25 nM) and TNF-*α* (5 ng/ml) for further experiments. Time-dependent results revealed that the combination of TP and TNF-*α* decreased the cell viability at 24-h time interval (Figures 1C,D). To confirm the results of cell viability experiments, protein expressions of cleaved caspase-3 and cleaved caspase-8 were analyzed using western blot. The results showed that TP/TNF-*α* cotreatment increased the expression of cleaved caspase-3 and cleaved caspase-8 (Figures 1E–H). These results confirmed that TP pretreatment sensitized AGS and MKN45 cells to TNF-*α* in gastric cancer.

### Triptolide Treatment Inhibited NF-κB–Mediated Prosurvival Signals Induced by TNF-*α*

**FIGURE 2:**
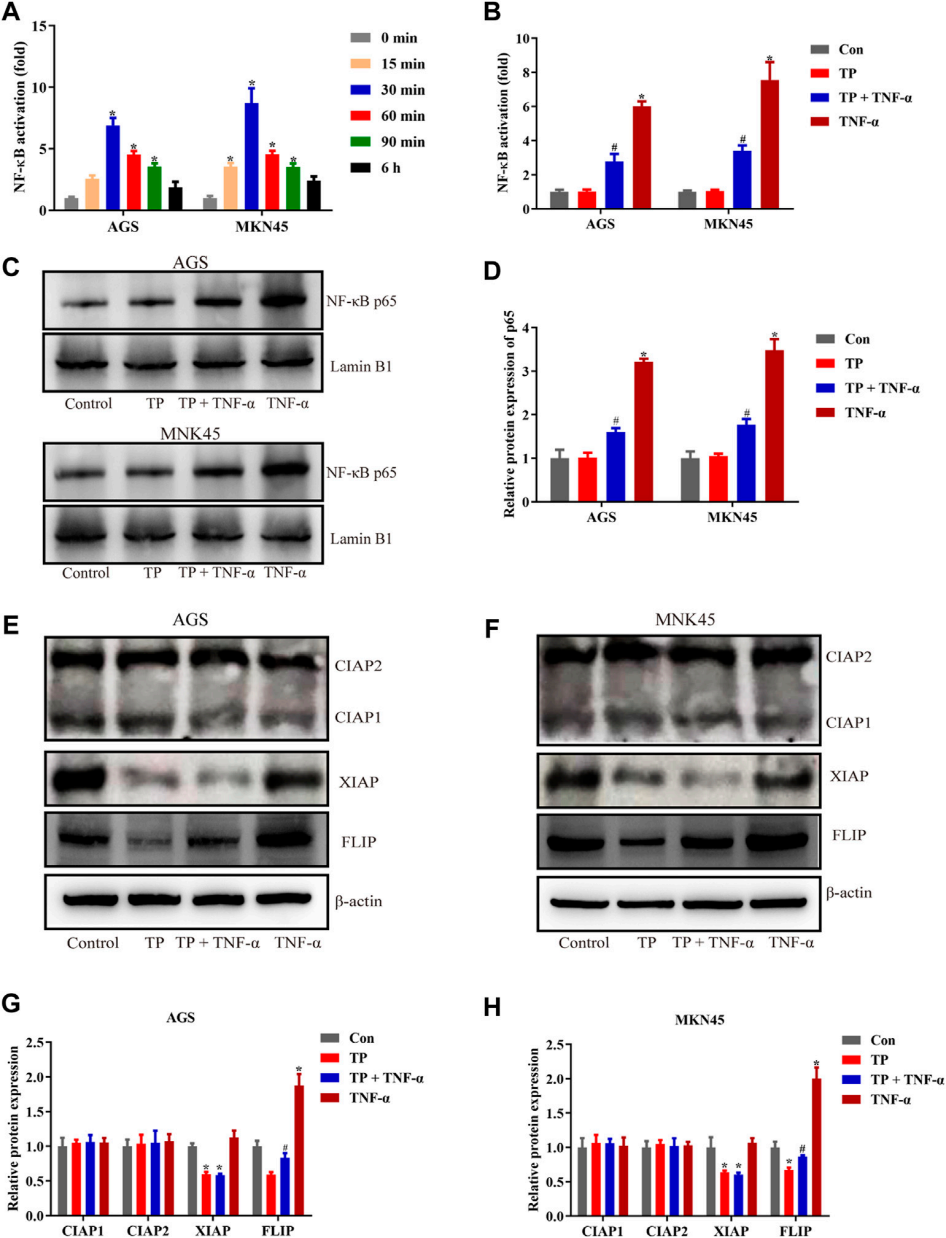
TP treatment inhibited NF-κB–mediated prosurvival signals induced by TNF-*α*. **(A)** Time-dependent NF-κB activation induced by TNF-*α* (5 ng/ml). **(B)** Relative NF-κB activity induced by TP (25 nM) and TNF-*α* (5 ng/ml), 30 min after TNF-*α* treatment. **(C–D)** Representative western blots and relative intensity of protein bands of NF-κB p65 nuclear protein, with Lamin B1 as the loading control, 30 min after TNF-*α* (5 ng/ml) treatment. **(E–H)** Representative western blots and relative intensity of protein bands of CIAP1, CIAP2, XIAP, and FLIP in AGS and MKN45 cells treated with TP (25 nM) and TNF-*α* (5 ng/ml), 24 h after TNF-*α* application, with *β*-actin as the loading control. Results were expressed as mean ± SEM, and statistical analysis was performed using one-way ANOVA or two-way ANOVA followed by Tukey’s multiple comparison test. *^, #^ *p* < 0.05 (*n* ≥ 3). *p compared with the control group and ^#^ *p* compared with the TNF-*α*-treated group.

It has been widely accepted that TNF-*α* is the inducer of apoptosis and necroptosis (Pasparakis and Vandenabeele, 2015; Peltzer et al., 2016). However, under a physiological state, TNF-*α* induces cell survival instead of cell death because of the checkpoints in the TNF-*α*-TNF-R1 pathway. Several checkpoints determine cell fate, and NF-κB–mediated prosurvival signals are one of them (Annibaldi and Meier, 2018). To determine the NF-κB activity, both cell lines were treated with TNF-*α* (5 ng/ml) for different time intervals. The result illustrated that NF-κB activity was increased in a time-dependent manner, restored to normal at the 6-h time interval, and peaked at the 30-min time interval (Figure 2A). Based on these observations, NF-κB activity was determined 30 min after TNF-*α* stimulation. The result in Figure 2B showed that TP/TNF-*α* cotreatment decreased NF-κB activity compared to TNF-*α* treatment alone, indicating that TP pretreatment inhibited the activity of NF-κB induced by TNF-*α*. Similar results were obtained after the detection of the protein level of NF-κB p65 (Figures 2C,D). NF-κB meditated prosurvival function depends on the expression of several prosurvival proteins. Thus, we detected the expressions of prosurvival proteins related to NF-κB, including CIAP1, CIAP2, XIAP, and FLIP. The results revealed that TP and TNF-*α* had little effect on the expressions of CIAP1 and CIAP2. In contrast, XIAP and FLIP protein expressions were decreased in the TP/TNF-*α* cotreatment group compared to the TNF-*α* treatment alone, and TNF-*α* induced the upregulation of FLIP in both the cell lines (Figures 2E–H). However, previous results implied that CIAP1, CIAP2, and XIAP cooperated to maintain embryonic development and protected cells from TNF-*α*–induced cell death (Moulin et al., 2012). Thus, we excluded the role of XIAP in TP/TNF-*α*–induced apoptosis in gastric cancer and supposed that FLIP might play an essential role in TP/TNF-*α*–induced apoptosis in AGS and MKN45 cells.

### Overexpression of FLIP Protected AGS and MKN45 Cells From TP/TNF-*α*–Induced Apoptosis

**FIGURE 3:**
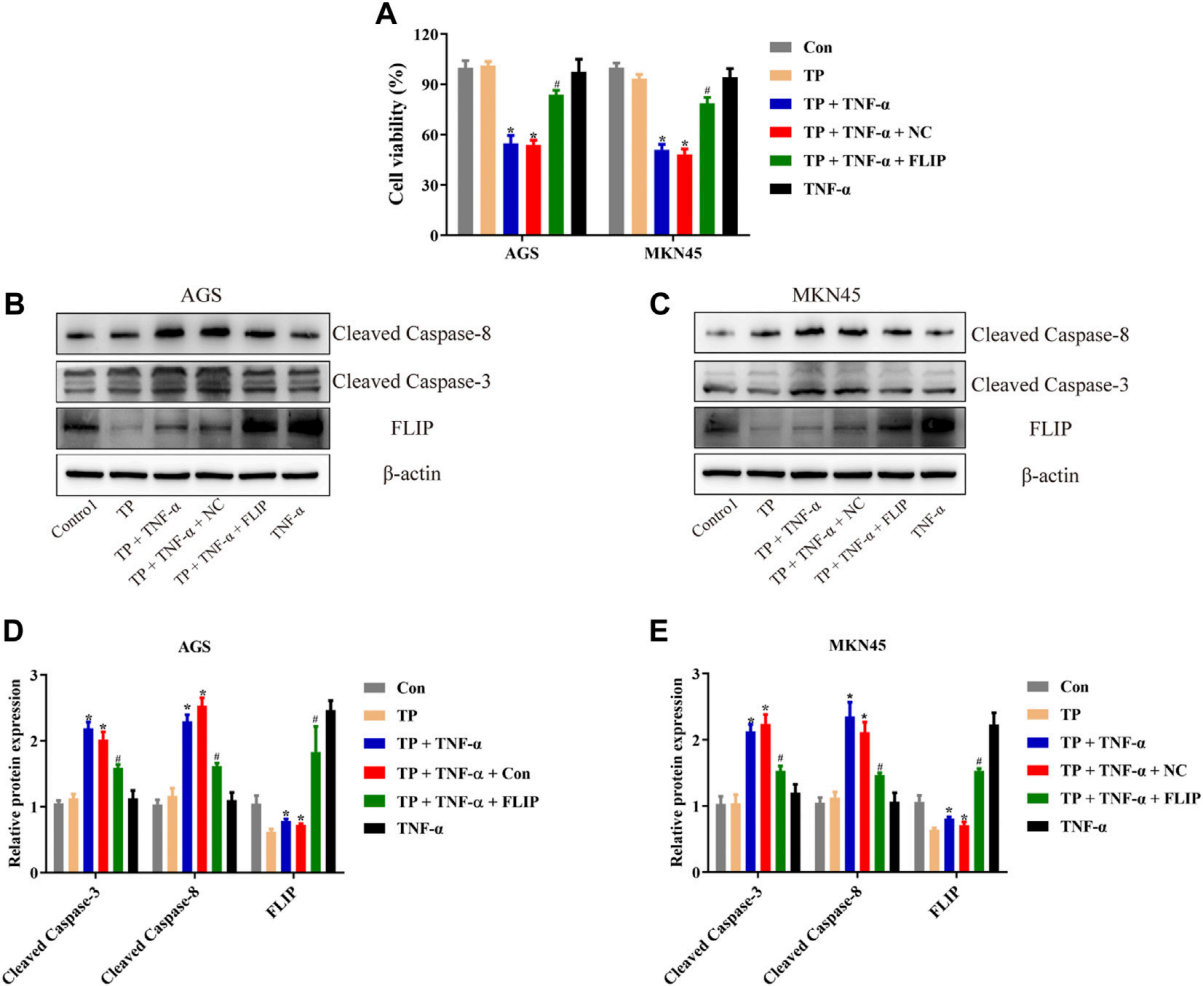
Overexpression of FLIP protected AGS and MKN45 cells from TP/TNF-*α*–induced apoptosis. **(A)** Cell viability of AGS and MKN45 cells transfected with a plasmid carrying FLIP sequence or its negative control with subsequent treatment with TP (25 nM) and TNF-*α* (5 ng/ml) for 24 h. **(B–E)** Representative western blots and relative intensity of protein bands of cleaved caspase-3, cleaved caspase-8, and FLIP in AGS and MKN45 cells transfected with FLIP plasmid and treated with TP (25 nM) and TNF-*α* (5 ng/ml), 24 h after TNF-*α* application, with *β*-actin as the loading control. Results were expressed as mean ± SEM, and statistical analysis was performed using two-way ANOVA followed by Tukey’s multiple comparison test. *^, #^ *p* < 0.05 (*n* ≥ 3). *p compared with the control group and ^#^ *p* compared with TP/TNF-*α*/NC-treated group.

To evaluate the function of FLIP in TP/TNF-*α*–induced apoptosis in AGS and MKN45 cells, both cell lines were transfected with the FLIP expression plasmid. The results showed that FLIP overexpression significantly increased the cell viability (Figure 3A). Relative protein expressions of cleaved caspase-3, cleaved caspase-8, and FLIP also revealed that FLIP overexpression protected the cells from TP/TNF-*α*–induced apoptosis (Figures 3B–E). These results showed that NF-κB–mediated FLIP expression had a protective role in TP/TNF-*α*–induced apoptosis.

### LncRNA H19 Acted as the Upstream Component of the NF-κB Pathway in TP/TNF-*α*–Stimulated AGS and MKN45 Cells

**FIGURE 4:**
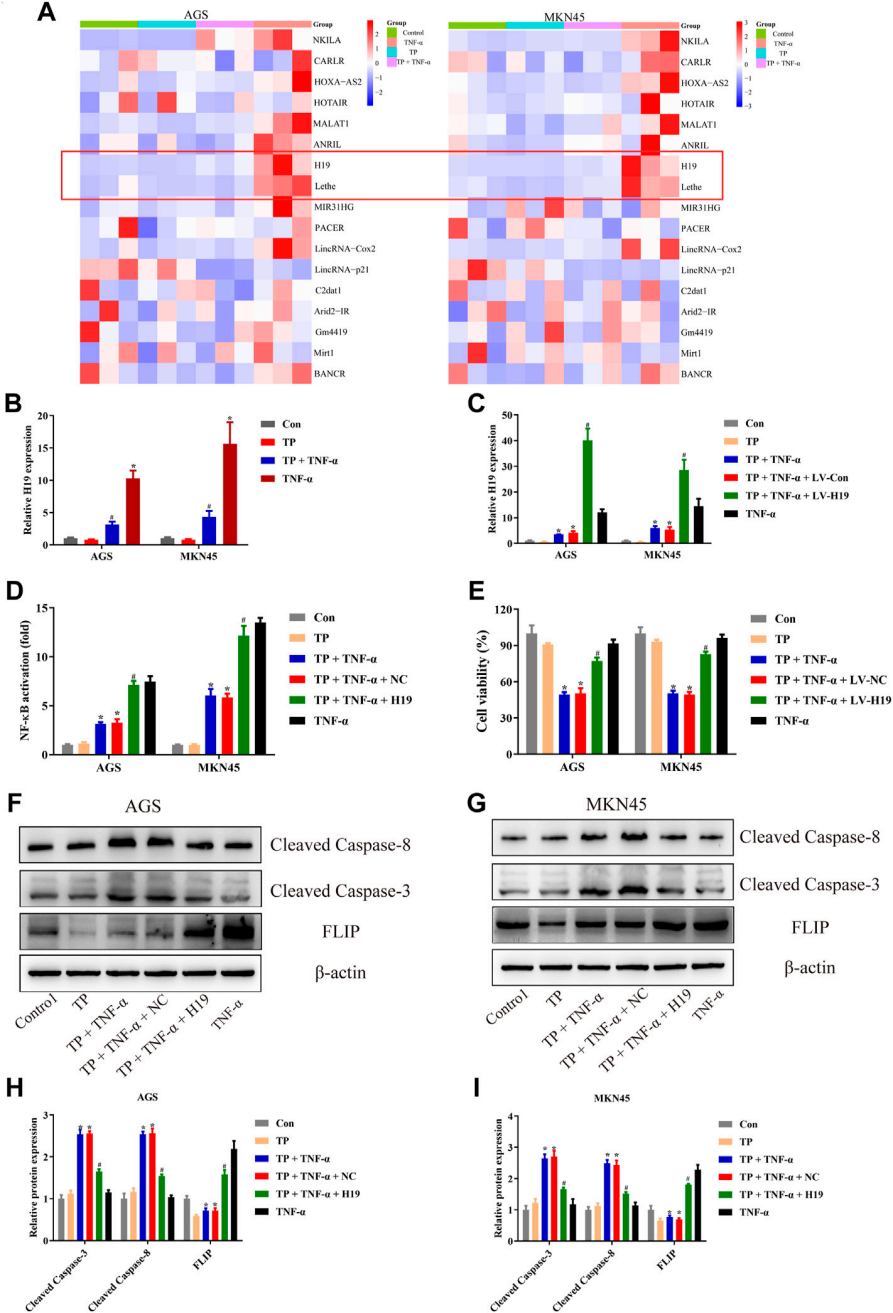
LncRNA H19 acted as the upstream component of the NF-κB pathway in TP/TNF-*α*–stimulated AGS and MKN45 cells. **(A)** NF-κB-related lncRNA was detected with qPCR 30 min after TNF-*α* stimulation. **(B)** Relative mRNA expression of H19 on cells treated with TP (25 nM) and TNF-*α* (5 ng/ml) for 24 h. **(C)** Relative mRNA expression of H19 on cells transfected with a plasmid carrying H19 sequence or its negative control treated with TP (25 nM) and TNF-*α* (5 ng/ml) for 24 h. **(D)** Relative NF-κB activity of cells transfected with H19 plasmid and treated with TP (25 nM) and TNF-*α* (5 ng/ml), 30 min after TNF-*α* treatment. **(E)** Cell viability of AGS and MKN45 cells transfected with a plasmid carrying H19 sequence or its negative control and treated with TP (25 nM) and TNF-*α* (5 ng/ml) for 24 h. **(F–I)** Representative western blots and relative intensity of protein bands of cleaved caspase-3, cleaved caspase-8, and FLIP in AGS and MKN45 cells transfected with H19 plasmid and treated with TP (25 nM) and TNF-*α* (5 ng/ml), 24 h after TNF-*α* application, with *β*-actin as the loading control. Results were expressed as mean ± SEM, and statistical analysis was performed using two-way ANOVA followed by Tukey’s multiple comparison test. *^, #^ *p* < 0.05 (*n* ≥ 3). *p compared with the control group and ^#^ *p* compared with TP/TNF-*α*/NC-treated group.

Numerous studies implied that abnormal expression of lncRNA participated in the tumorigenesis and tumor progression of multitype cancer cells, including gastric cancer (Yu and Rong, 2018; Yuan L. et al., 2020; Wei et al., 2020). The regulation of lncRNAs and their bioactivity has attracted researchers’ attention for their function in cancer diagnosis, prognosis, as well as chemotherapy (Wei et al., 2020). To identify the unrevealed lncRNA that might participate in TP/TNF-*α*–induced antigastric cancer effect, we screened the generally accepted lncRNAs that related to NF-κB activation (Gupta et al., 2020). Among them, we found that lncRNA H19 and Lethe were both increased by TNF-*α* in AGS and MKN45 cells (Figure 4A). LncRNA H19 was recognized as the oncogene of gastric cancer. Existing scientific research pointed out that overexpression of H19 promoted gastric cancer cell invasion and migration, while the inhibition of H19 inhibited gastric cancer cell growth (Liu et al., 2016; Zhang et al., 2017; Gan et al., 2019). As H19 was reported to be the upstream member of the NF-κB pathway upon TNF-*α* stimulation, we speculated that H19 might play an essential role in TP/TNF-*α*–induced apoptosis in gastric cancer cells. Researchers found that TNF-*α* treatment significantly enhanced the expression of H19, while TP pretreatment inhibited this process (Figure 4B). To identify whether H19 acted as the upstream component of the NF-κB under the stimulation of TNF-*α* in gastric cancer cells, H19 siRNA was transfected into AGS and MKN45 cells before TNF-*α* treatment. qPCR analysis revealed that two NF-κB targeting genes, *NFKBIA* and *TNFAIP3*, were significantly increased in TNF-*α* treated cells, while H19 siRNA obstructed this process (Supplementary Figures S1A,B). Moreover, H19 siRNA pretreatment decreased the translocation of p65 from the cytoplasm into the nucleus in TNF-*α*-treated AGS and MKN45 cells (Supplementary Figures S1C,D). We also transfected the H19 plasmid into these two cell lines. The results revealed that cells transfected with H19 overexpression plasmid showed a relative increase in H19 expression and significantly increased the activity of NF-κB (Figures 4C,D). Moreover, H19 overexpression increased the cell viability of the cells treated with TP/TNF-*α* (Figure 4E). Western blot results also confirmed that H19 overexpression exhibits a cytoprotective effect in both cell lines as cleaved caspase-3 and cleaved caspase-8 levels were decreased, while the FLIP level was increased in TP/TNF-*α*/H19 group (Figures 4F–I). These results explained that TP–induced sensitivity of AGS and MKN45 cells to TNF-*α* was dependent on H19.

### H19 Showed Direct Binding to miR-204-5p

**FIGURE 5:**
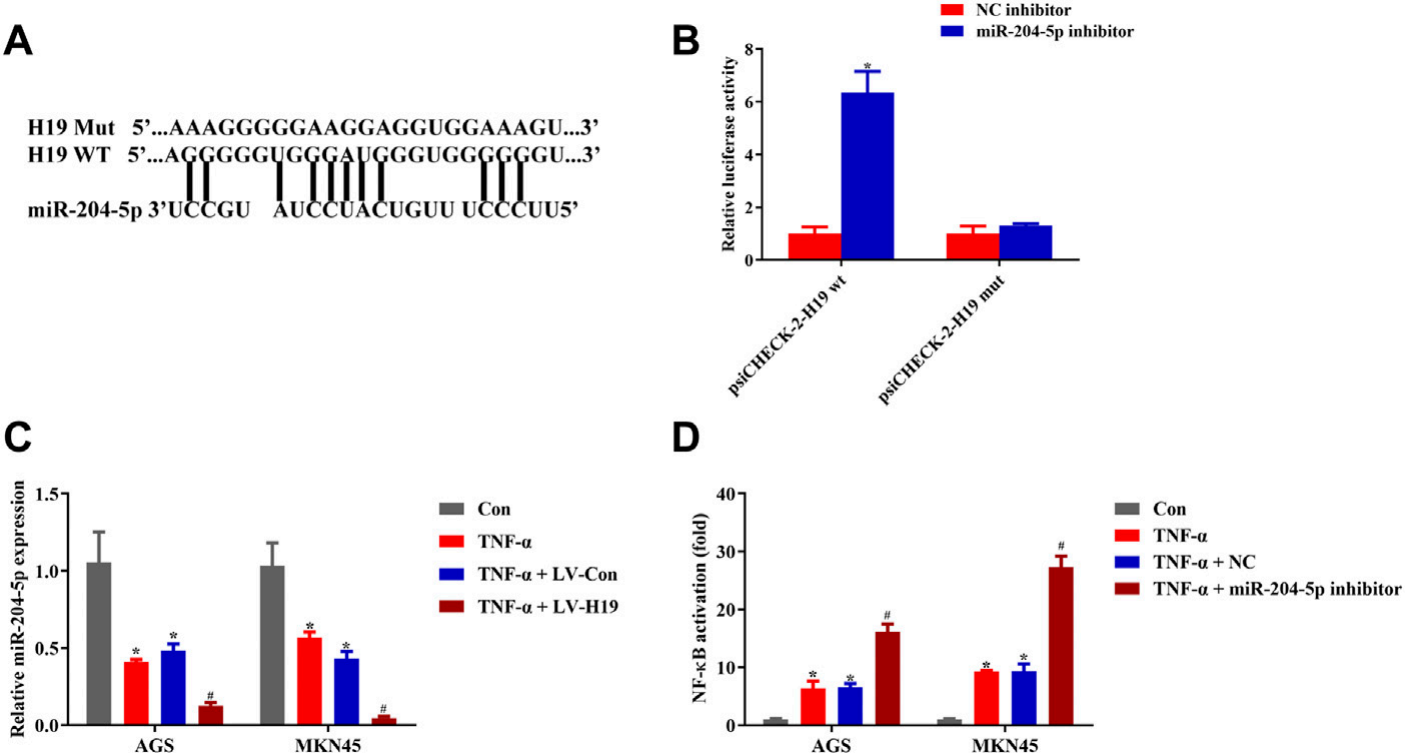
H19 showed direct binding to miR-204-5p. **(A)** Predicted binding sites between miR-204-5p and H19. **(B)** Dual-luciferase reporter assay of psiCHECK-2-H19 wt and psiCHECK-2-H19 mut HEK 293T cell cotransfected with miR-204-5p inhibitor or its negative control. **(C)** Relative mRNA expression of miR-204-5p in cells transfected with H19 or its negative control and treated with TNF-*α* (5 ng/ml) for 24 h. **(D)** Relative NF-κB activity of cells transfected with miR-204-5p inhibitor or its negative control and treated with TNF-*α* (5 ng/ml) for 24 h. Results were expressed as mean ± SEM, and statistical analysis was performed using two-way ANOVA followed by Tukey’s multiple comparison test. *^, #^ *p* < 0.05 (*n* ≥ 3). *p compared with the control group and ^#^ *p* compared with TNF-α/LV-Con or TNF-*α*/NC-treated group.

To identify how H19 regulated NF-κB activity upon TNF-*α* stimulation, we screened the candidate miRNA that related to NF-κB and selected the most suitable eight miRNAs for our study (Mirzaei et al., 2021). We used RNAhybrid to predict the binding of the selected miRNAs to H19, finding that miR-204-5p binding to H19 needed the minimum MFE (Supplementary Material). According to the prediction, there is a putative binding site of miR-204-5p in H19 (Figure 5A). Dual-luciferase reporter assay conducted in HEK293T cells implied that miR-204-5p inhibitor significantly increased H19 WT luciferase activity while having little effect on H19 Mut cells (Figure 5B). In addition, TNF-*α* treatment inhibited the expression of miR-204-5p, and transfection of H19 plasmid into both cell lines decreased the relative miR-204-5p expression (Figure 5C). NF-κB activity assay revealed that the miR-204-5p inhibitor decreased the upregulation of NF-κB induced by TNF-*α* (Figure 5D). These results revealed that H19 inhibited the expression of miR-204-5p, which acted as the activator of the upstream pathway of NF-κB upon TNF-*α* stimulation in AGS and MKN45 cells.

### miR-204-5p Inhibitor Protected AGS and MKN45 Cells From Triptolide/TNF-*α*–Induced Apoptosis

**FIGURE 6:**
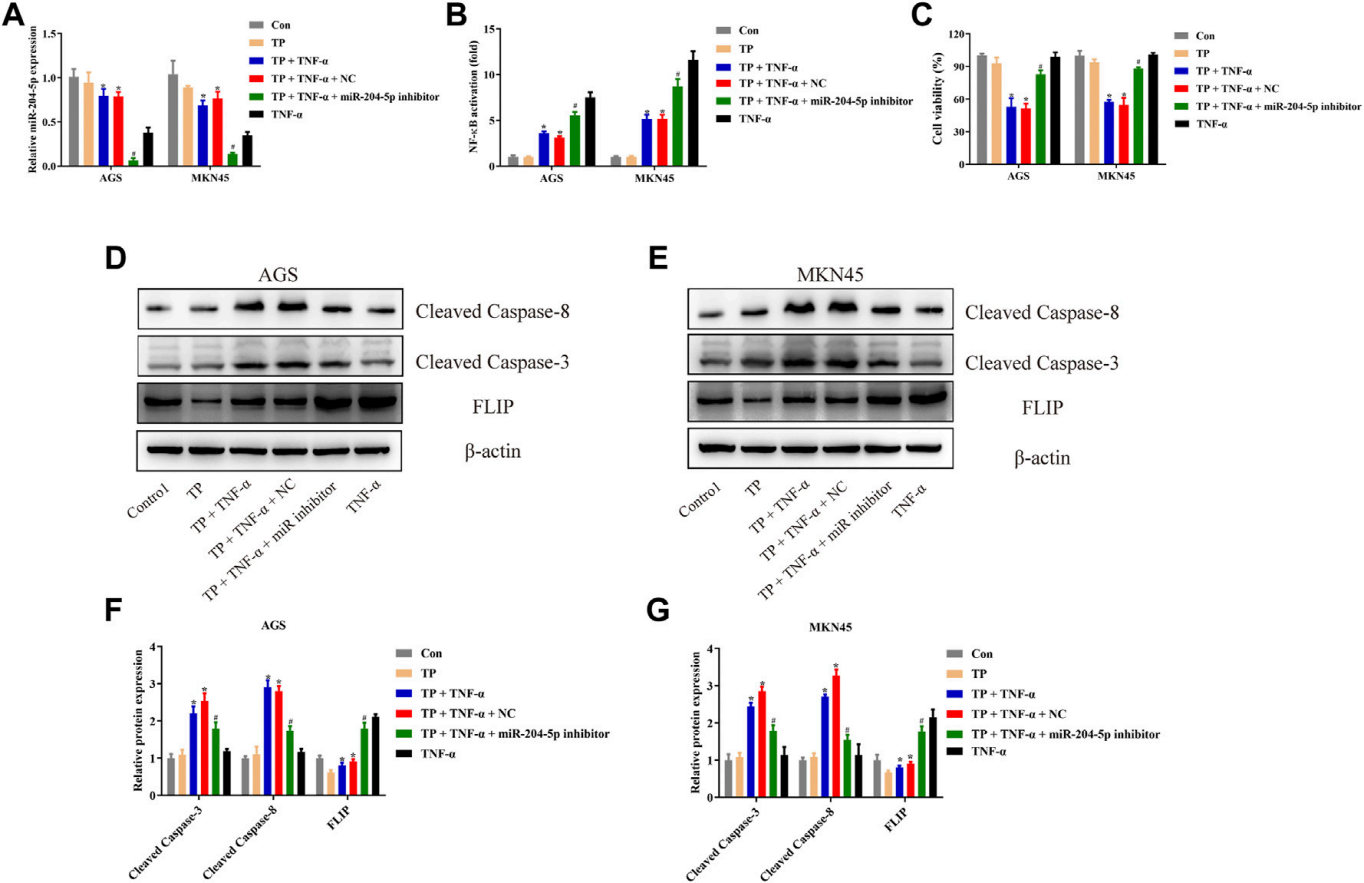
miR-204-5p inhibitor protected AGS and MKN45 cells from TP/TNF-*α*–induced apoptosis. **(A)** Relative mRNA expression of miR-204-5p in cells transfected with miR-204-5p inhibitor and treated with TP (25 nM) and TNF-*α* (5 ng/ml) for 24 h. **(B)** Relative NF-κB activity of cells transfected with miR-204-5p inhibitor and treated with TP and TNF-*α* for 30 min. **(C)** Cell viability of AGS and MKN45 cells transfected with miR-204-5p inhibitor and treated with TP (25 nM) and TNF-*α* (5 ng/ml) for 24 h. **(D–G)** Representative western blots and relative intensity of protein bands of cleaved caspase-3, cleaved caspase-8, and FLIP in AGS and MKN45 cells transfected with miR-204-5p inhibitor and treated with TP (25 nM) and TNF-*α* (5 ng/ml), 24 h after TNF-*α* application, with *β*-actin as the loading control. Results were expressed as mean ± SEM, and statistical analysis was performed using two-way ANOVA followed by Tukey’s multiple comparison test. *^, #^ *p* < 0.05 (*n* ≥ 3). *p compared with the control group and ^#^ *p* compared with TP/TNF-*α*/miR-204-5p inhibitor-treated group.

To investigate the role of miR-204-5p in TP/TNF-*α*–induced apoptosis in gastric cancer cells, AGS and MKN45 cells were additionally treated with miR-204-5p inhibitor or its NC. According to the results in Figures 6A–C, the miR-204-5p inhibitor group showed a decrease in the relative expression of miR-204-5p and an increase in NF-κB activity and cell viability in TP/TNF-*α* treated AGS and MKN45 cells. Western blot results also illustrated that miR-204-5p inhibitor treatment had a cytoprotective role by decreasing the protein expressions of cleaved caspase-3 and cleaved caspase-8 and increasing the level of FLIP (Figures 6D–G). These results firmly proved that miR-204-5p was the upstream member of the NF-κB pathway and was negatively correlated to NF-κB activation.

### Triptolide/TNF-*α* Inhibited the Growth of AGS and MKN45 Cells *in vivo*

**FIGURE 7:**
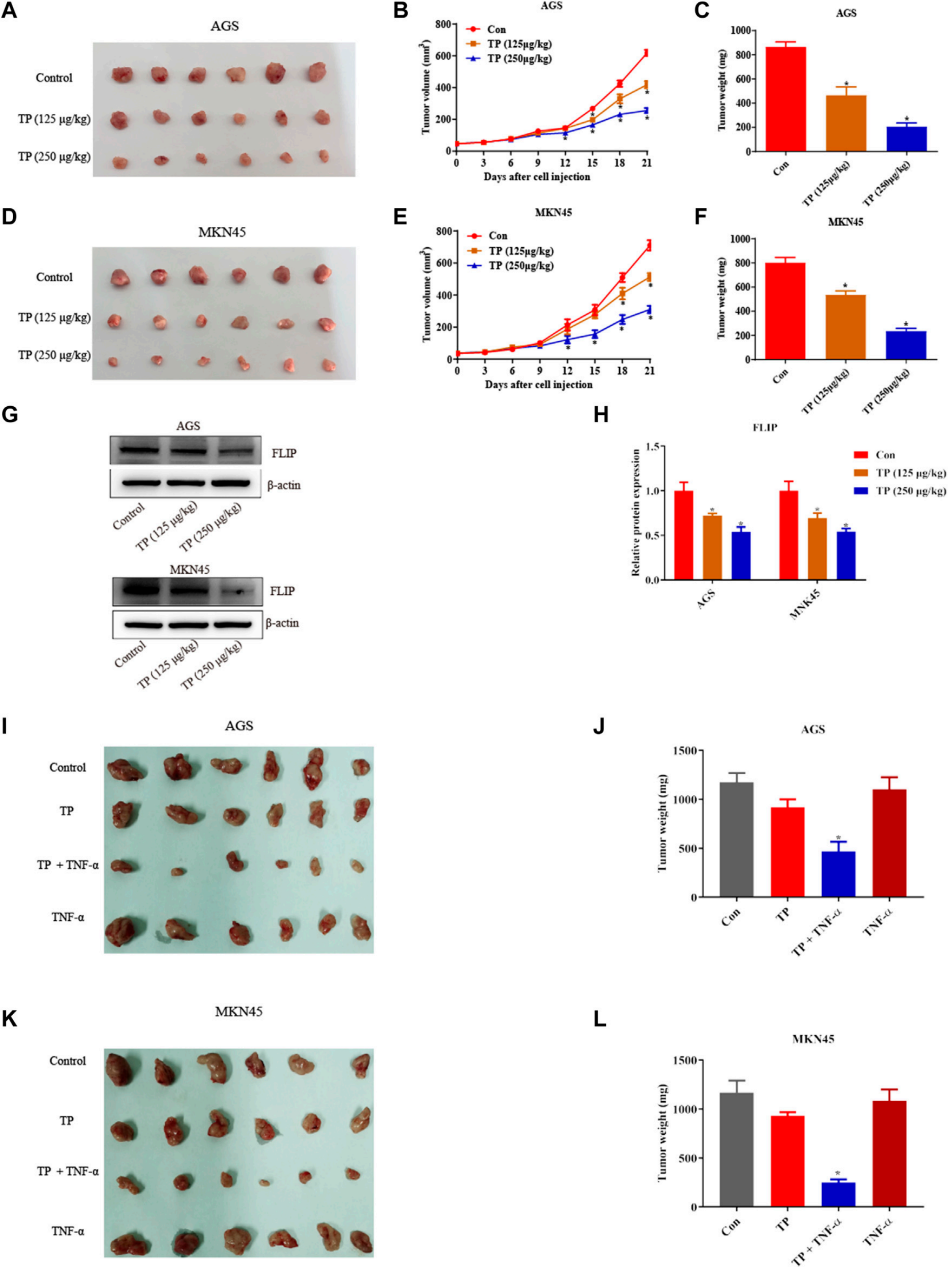
TP inhibited the growth of AGS and MKN45 cells *in vivo*. **(A)** Images of tumor size when nude mice were subcutaneously injected with AGS cells and treated with TP (125 μg/kg or 250 μg/kg) for 3 weeks. **(B–C)** Tumor volume and weight of mice injected with AGS cells treated with TP. **(D)** Images of tumor size when nude mice were subcutaneously injected with MKN45 cells and treated with TP (125 μg/kg or 250 μg/kg) for 3 weeks. **(E–F)** Tumor volume and weight of mice injected with MKN45 cells treated with TP. **(G–H)** Representative western blots and relative intensity of protein bands of FLIP in mice injected with AGS and MKN45 cells and treated with TP (125 μg/kg or 250 μg/kg). **(I–L)** Images of tumor size and weight of nude mice injected with TP (125 μg/kg) and TNF-*α* (5 μg/kg) for 3 weeks. Results were expressed as mean ± SEM, and statistical analysis was performed using one-way ANOVA or two-way ANOVA followed by Tukey’s multiple comparison test. *p < 0.05 (*n* ≥ 3). *p compared with the control group.

Lastly, the antigastric cancer effects of TP were investigated *in vivo*. Mice were subcutaneously injected with AGS cells and MKN45 cells and were treated with different doses of TP (125 μg/kg and 250 μg/kg) for 21 days. Two doses of TP inhibited the tumor volume and tumor weight of AGS and MKN45 cells in a dose-dependent manner (Figures 7A–F). Western bolt analysis revealed that TP inhibited the expression of FLIP at both doses (Figures 7G,H). Then, we treated mice with TP (125 μg/kg) for 3 weeks and TNF-*α* (5 μg/kg) twice a week. Three weeks after TP administration, we found that TNF-*α* alone has little effect on the tumor weight and volume. However, TP/TNF-*α* cotreatment dramatically inhibited the tumor growth of AGS and MKN45 cells and firmly proved that TP treatment increased the sensitivity of AGS and MKN45 cells to TNF-*α in vivo* (Figures 7I–L).

## Discussion

TP and its derivatives, such as Minnelide and PG490-88, attracted researchers’ attention because of their multiple pharmacological activities. The antitumor activity of TP is associated with the inhibition of tumor cell growth, induction of tumor cell death, or cell cycle arrest in diverse types of cancers, such as breast cancer, acute myeloid leukemia, lung cancer, ovarian cancer, neuroblastoma, prostate cancer, osteosarcoma, and gastric cancer (Pigneux et al., 2008; Krosch et al., 2013; Rivard et al., 2014; Shao et al., 2014; Jiang et al., 2016; Isharwal et al., 2017; Jiang et al., 2018). However, the application of TP and its derivatives was restricted due to their side effects. Structural modifications, reduction in the dose, or improving the delivery system of TP may be the better choices for therapeutic uses of TP. Previous research found that TP increased the sensitivity of hepatocytes upon TNF-*α* exposure, and we wanted to utilize this characteristic of TP to increase its antitumor efficiency (Yuan et al., 2019). This study was designed to explore whether TP increased the sensitivity of gastric cancer cells to the TNF-*α* stimulation and tried to find the mechanisms behind it.

Experimental results of Figures 1A–D showed that TP pretreatment sensitized gastric cancer cell lines to TNF-*α* in a time-dependent manner, and we selected nontoxic doses of TP and TNF-*α* for further experiments. In most cases, cells exposed to the low dose of TNF-*α* did not experience cell death until the checkpoints of the TNF-*α* pathway became out of control, keeping the NF-κB–mediated prosurvival signal indispensable. Next, mechanistic studies revealed that TP inhibited NF-κB–mediated FLIP expression that was upregulated by the stimulation of TNF-*α*, and this observation was the same as reported in the previous study (Yuan Z. et al., 2020). However, the mechanism behind TP–induced inhibition of NF-κB–mediated FLIP expression leading to an increase in gastric cancer cell sensitivity to TNF-*α* stimulation remained unclear.

Some experimental reports revealed that lncRNA may not only participate in the progress of gastric cancer but also control the activity of NF-κB (Lin et al., 2014; Sun et al., 2017). After screening the lncRNAs, we found that H19 might be the appropriate candidate for our study. H19, expressed only on maternal allele and imprinted in both humans as well as mice, was the first lncRNA gene to be discovered. It has been reported that an elevated level of H19 is present in gastric cancer and bladder cancer, while the decreased level is present in hepatocellular carcinoma, indicating that H19 has both tumor suppressor as well as oncogenic properties (Iizuka et al., 2004; Yang et al., 2012; Song et al., 2013b; Luo et al., 2013). Overexpression of H19 in kidney tumor cells reduced the growth rate along with the absence of neoplasm in mice, suggesting the tumor suppressor capacity of H19 in renal carcinogenesis (Matouk et al., 2005). A scientific study on the effect of H19 on gastric cancer showed that the H19 level was significantly elevated in AGS gastric cancer cell line. This increased level of H19 led to an increase in cell proliferation, while the use of H19 siRNA caused cell death in AGS cells (Yang et al., 2012). Another experimental study on gastric cancer illustrated that H19 expression was markedly increased in gastric tumor tissues compared with noncancer tissues. The increased level of H19 in gastric cancer tissues and gastric cancer cell lines suggested a main role of H19 in gastric cancer pathology (Song et al., 2013a). Based on these scientific developments, H19 has proven itself a diagnostic and therapeutic marker in various cancers, and its main role in carcinogenesis has been established. Moreover, it has been reported that H19 activates NF-κB *via* TAK1 phosphorylation and promotes gastric cancer cell growth *via* NF-κB activation (Zhang et al., 2019; Yang et al., 2020a). To identify whether H19 is the upstream member of the NF-κB pathway in AGS and MKN45 cells, we transfected the H19 plasmid into these cell lines. The results revealed that cells transfected with H19 overexpression plasmid showed a relative increase in H19 expression and a significant increase in the activity of NF-κB (Figure 4D). H19 overexpression also increased the cell viability after TP/TNF-*α* treatment (Figure 4E). Our experimental design for the first time depicted that H19 is the upstream member of NF-κB signaling in gastric cancer cells upon TNF-*α* stimulation. However, no existing research reveals that H19 deficit mice are sensitive to LPS or TNF-*α*, which needs to be proved in future studies.

It has been revealed that an abnormal level of miRNAs is involved in the development of neoplasm, and some of them are associated with gastric carcinogenesis by regulating cancer-related genes. Moreover, they also prove to be important cancer biomarkers (Li et al., 2011; Shin and Chu, 2014). Most of the miRNAs are engaged in critical cellular processes, including proliferation and invasion, by controlling the posttranscriptional level of target gene expressions. For instance, miR-589 remarkably promoted gastric cancer proliferation and invasion through miR-589-P13/AKT-c-Jun signaling (Zhang et al., 2018). MiRNA-21 enhanced gastric cancer cell growth by regulating PEG2 (Qi et al., 2018). To find out the physiological mechanism of H19, we used RNAhybrid to predict the binding of H19 to biochemically suitable miRNA and found that miR-204-5p was the ideal candidate. Our results found that transfection with H19 overexpression plasmid inhibited the miR-204-5p level upon TNF-*α* stimulation in AGS and MKN45 cells (Figure 5). In addition, the miR-204-5p inhibitor increased the activity of NF-κB and cell viability upon TP/TNF-*α* treatment. Western blot results also showed a decrease in the protein expression of proapoptotic cleaved caspase-3 and cleaved caspase-8 proteins and an increase in the protein expression of the prosurvival protein, FLIP (Figure 6). Other studies have also reported the frequent downregulation of miR-204-5p in other tumor cells, including colorectal cancer cells and head and neck squamous cell carcinoma, indicating a similar effect of miR-204-5p in carcinogenesis (Shuai et al., 2018; Zhuang Z. et al., 2020).

In this hypothesis, we proved that TP–induced sensitivity of AGS and MKN45 cells to the TNF-*α* treatment was dependent on H19/miR-204-5p/NF-κB signaling. It is known that the injection of TNF-*α via* the tail vein quickly distributes in multiple organs that may cause some side effects. We investigated the effect of cotreatment of TP and TNF-*α in vivo*; however, the distribution of TNF-*α* in the tissue is so quick that there is no difference in the level of TNF-*α* in the serum and tumor tissue groups after iv injection of TNF-*α* (10 μg/kg) into the tail vein, although there might be some differences in the levels of miR-204-5p and H19 between the TP/TNF-*α* and TNF-*α*-treatment groups. Moreover, iv injection of TNF-*α* into the tail vein slightly upregulated NF-κB target genes (compared with the published articles), *NFKBIA*, and *TNFAIP3*, which were observed to examine the effect of TNF-*α* on the tumor NF-κB activity (Supplementary Figure S2). Due to the rapid tissue distribution of TNF-*α*, intratumoral injection of TNF-*α* was administered *in vivo*. The result proved that TP/TNF-*α* inhibited the growth of AGS and MKN45 cells. Considering the poor accessibility of intratumoral injection, we supposed that exosome-based antibodies or other formulations for TNF-*α* delivery might be the optional plan (Probst et al., 2019a; Zhuang et al., 2020a). In addition, researchers proved that TNF-*α* delivery can enhance the antitumor activity of antibody-dependent cell–mediated cytotoxicity of an anti-Melanoma Immunoglobulin, peptide anticancer vaccine, and cancer cell membrane targeting therapy (Probst et al., 2019b; Murer et al., 2019; Zhuang et al., 2020b). We believe that new formulations focusing on the codelivery of TP and TNF-*α* nanomaterials for targeting tumor tissues can be a better solution for the antitumor effects of TP.

## Conclusion

**FIGURE 8:**
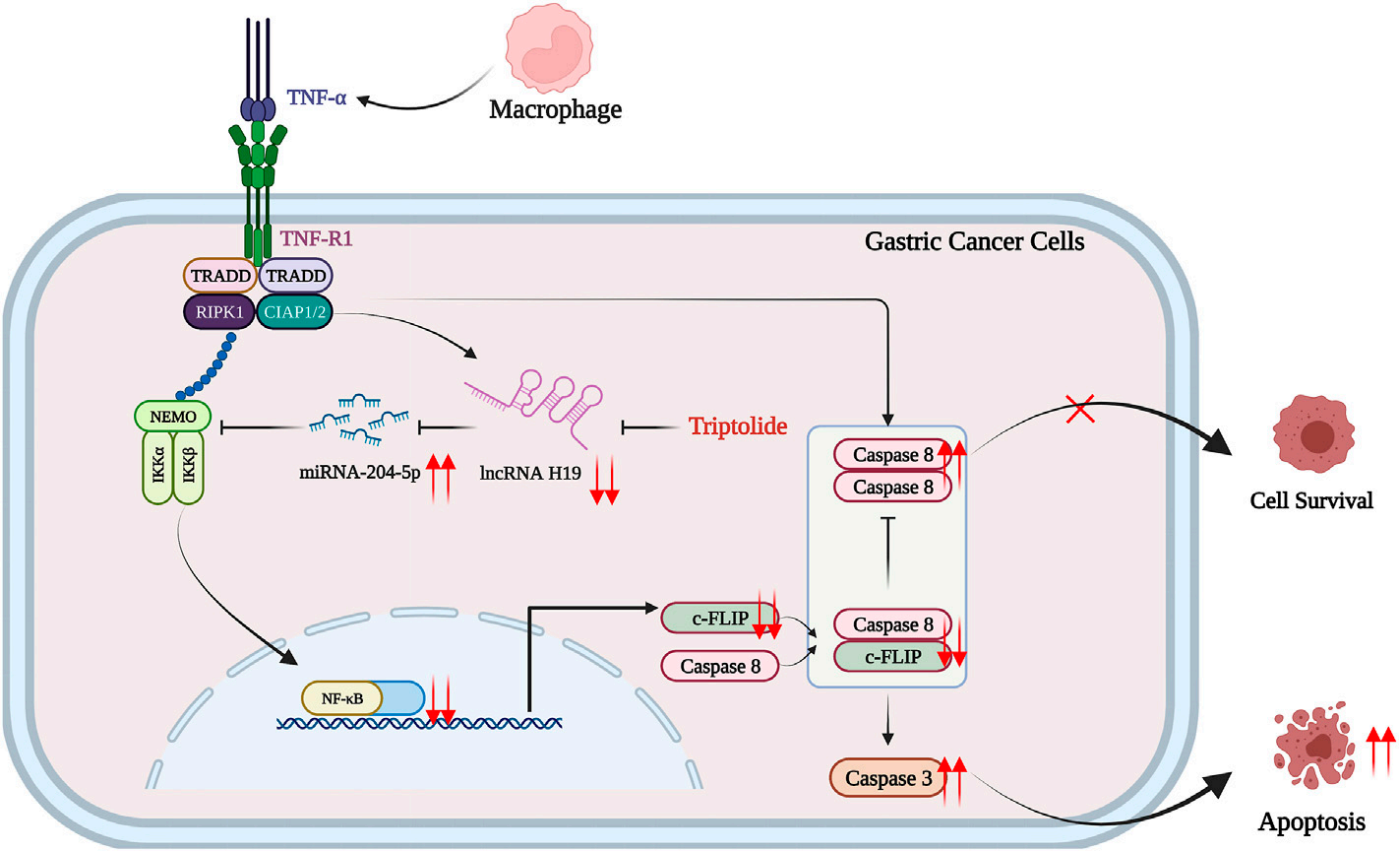
Schematic presentation indicating the suggested mechanisms by which TP/TNF-*α*–induced gastric cancer cell death. Under a physiological state, a low dose of TNF-*α* stimulates both cell survival signals *via* H19/miR-204-5p/NF-Κb/FLIP pathway and cell death signals *via* activating caspase-8. The upregulation of FLIP inhibits caspase-8-dependent apoptosis, and the consequence of a low dose of TNF-*α* is cell survival. However, TP pretreatment inhibited H19/miR-204-5p/NF-κB/FLIP signals, and the insignificant level of FLIP could not counteract the prodeath signals induced by TNF-*α*, leading to gastric cancer cell death. The figure was created using BioRender.com (Agreement number: IQ23Z4T0E3).

Pretreatment with a nontoxic dose of TP promoted apoptosis in AGS and MKN45 cells upon TNF-*α* treatment. The inhibition of FLIP by lncRNA H19 and miR-204-5p interaction lying upstream of NF-κB is indispensable for inflammatory sensitization (Figure 8). In addition, codelivery of TP and TNF-*α* nanomaterial might be the better solution for the antitumor effects of TP.

## Data Availability

The original contributions presented in the study are included in the article/Supplementary Material. Further inquiries can be directed to the corresponding authors.

## Supplementary Material

Supplementary Figure S1 Relative expression of the mRNA levels of NF-κB target genes NFKBIA and TNFAIP3 in AGS and MKN45 cells pretreated with H19 siRNA and stimulated with TNF-*α* for 30 min. **(C,D)** The protein level of NF-κB in AGS and MKN45 cells pretreated with H19 siRNA and stimulated with TNF-*α* for 30 min. Results were expressed as mean ± SEM, and statistical analysis was performed using two-way ANOVA followed by Tukey’s Multiple Comparison Test. *, ^#^ *p* < 0.05 (*n* ≥ 3). *P compared with the control group and #P compared with the TNF-*α*-siRNA-NC group.

Supplementary Figure S2 Serum **(A)** and tumor tissue **(B)** concentration of TNF-*α* in mice subcutaneously injected with AGS or MKN45 cells and treated with TP (125 μg/kg, ip) once a day and TNF-*α* (10 μg/kg, iv) once a week. **(C–F)** Relative mRNA expression of NF-κB target genes (NFKBIA and TNFAIP3), H19, and miR204-5p in mice treated with TP and TNF-*α*. Results were expressed as mean ± SEM, and statistical analysis was performed using Two-way ANOVA followed by Tukey’s multiple comparison test. *, ^#^ *p* < 0.05 (*n* ≥ 3). **p* compared with the control group and #P compared with the TNF-*α*-treated group.

## Abbreviations

CIAP1, Cellular inhibitor of apoptosis protein 1; CIAP2, Cellular inhibitor of apoptosis protein 2; FADD, Fas-associated protein with death domain; FLIP, Cellular FLICE-inhibitory protein; LPS, Lipopolysaccharide; lncRNA, Long Noncoding RNA; NFKBIA, NFKB Inhibitor Alpha; TP, Triptolide; TNF-*α*, Tumor necrosis factor-*α*; TNFAIP3, TNF Alpha–Induced Protein 3; TRAF2, TNF receptor-associated factor 2; XIAP, X-linked inhibitor of apoptosis protein.
