# Supplementary figures and images for: The Antigastric Cancer Effect of Triptolide is Associated With H19/NF-κB/FLIP Axis
Source: Front Pharmacol. 2022 Aug 30;13:918588. doi: 10.3389/fphar.2022.918588 (PMC9469193; doi:10.3389/fphar.2022.918588)

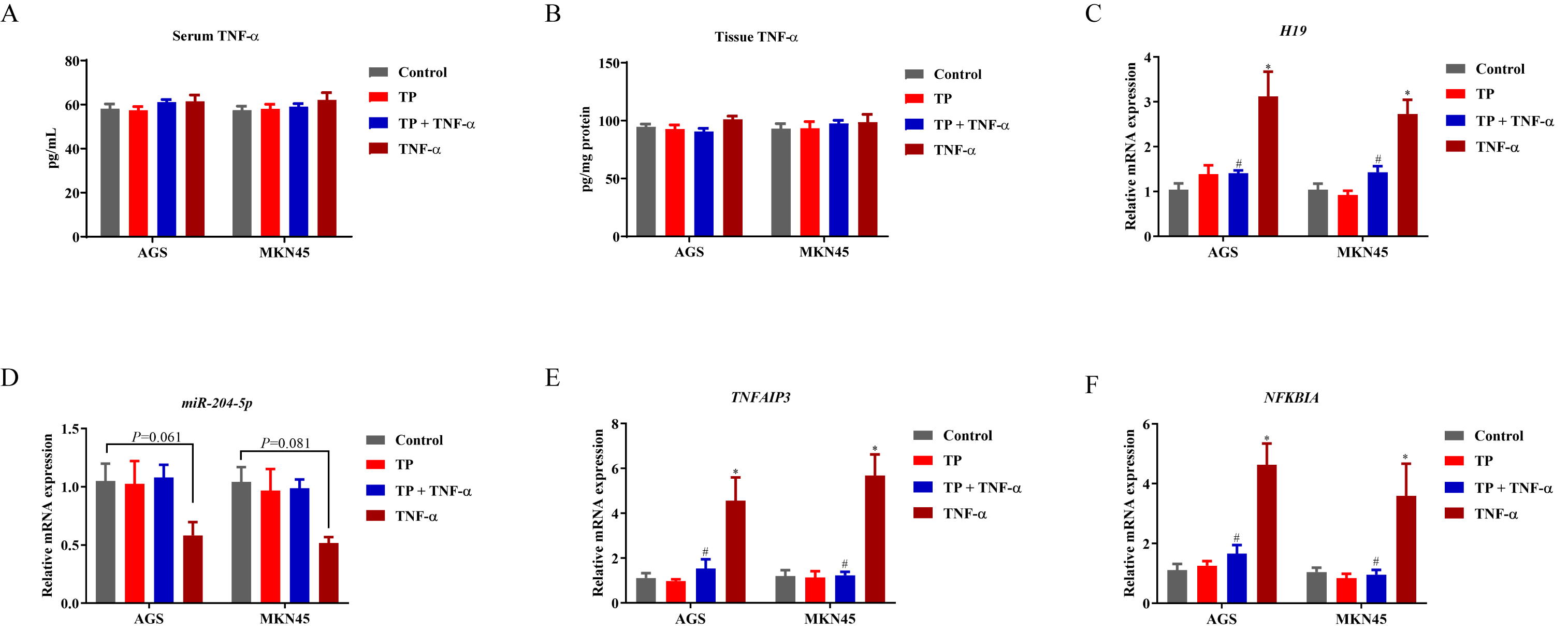

Supplement: Supplementary file 1 [file Image2.tif]

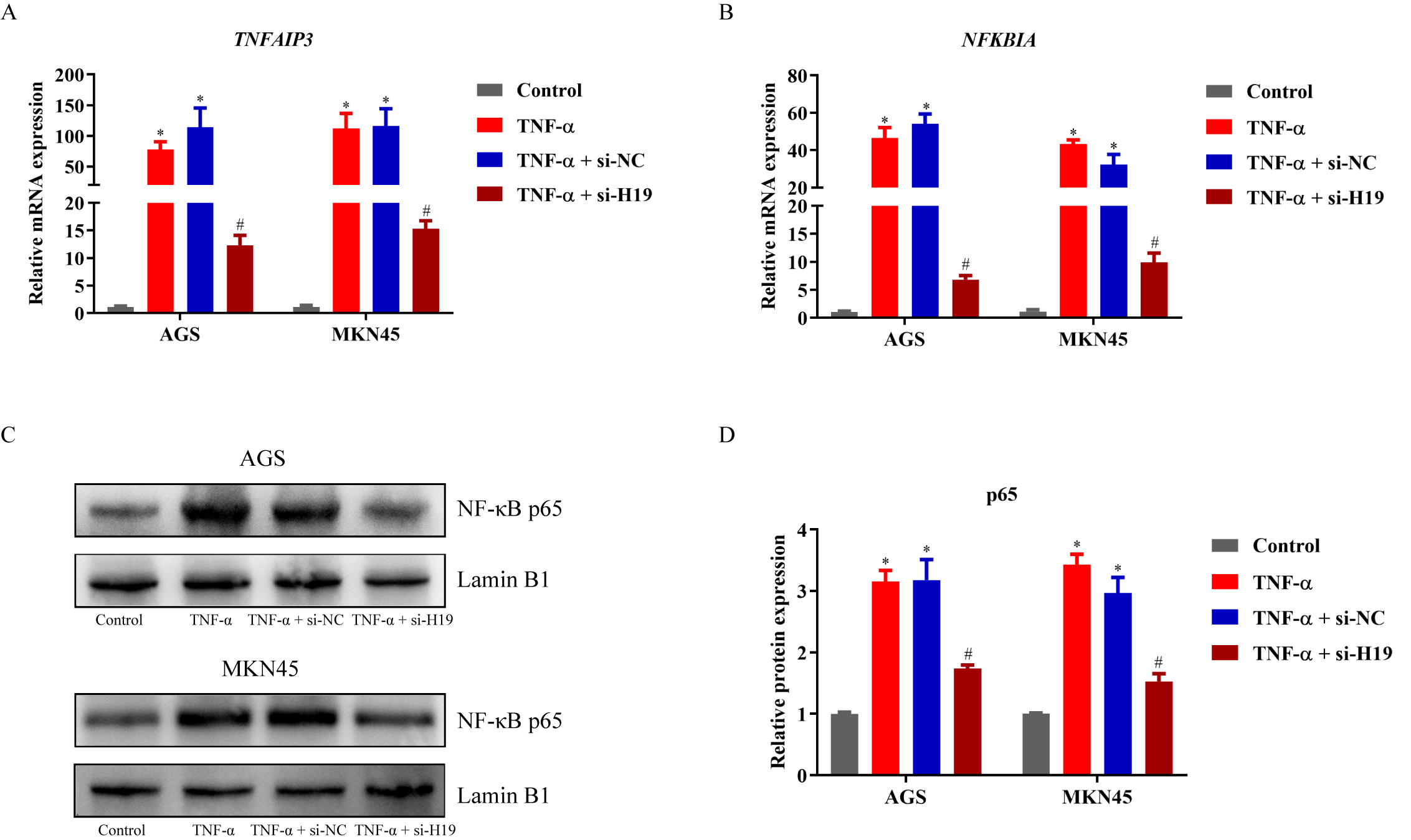

Supplement: Supplementary file 2 [file Image1.tif]
