# Supplementary material for: The Antigastric Cancer Effect of Triptolide is Associated With H19/NF-κB/FLIP Axis
Source: Front Pharmacol. 2022 Aug 30;13:918588. doi: 10.3389/fphar.2022.918588 (PMC9469193; doi:10.3389/fphar.2022.918588)

**>hsa-miR-532-5p MIMAT0002888**

CAUGCCUUGAGUGUAGGACCGU


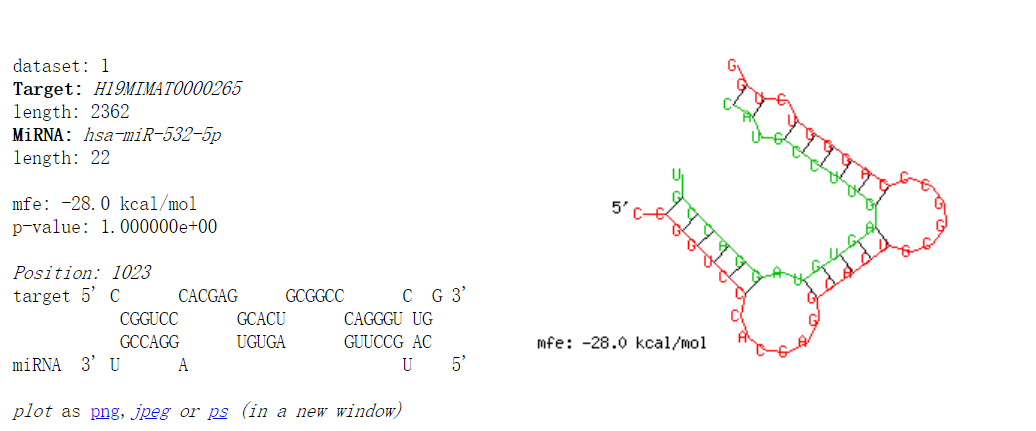


**>hsa-miR-92a-1-5p MIMAT0004507**

AGGUUGGGAUCGGUUGCAAUGCU


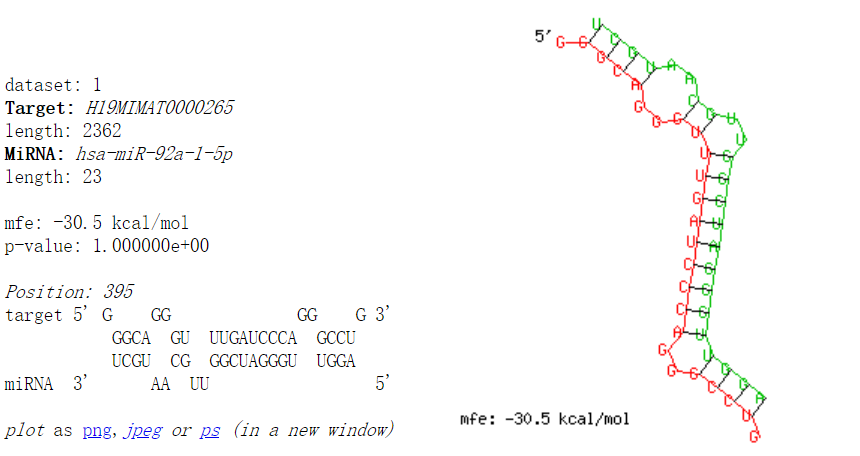


**>hsa-miR-508-3p MIMAT0002880**

UGAUUGUAGCCUUUUGGAGUAGA


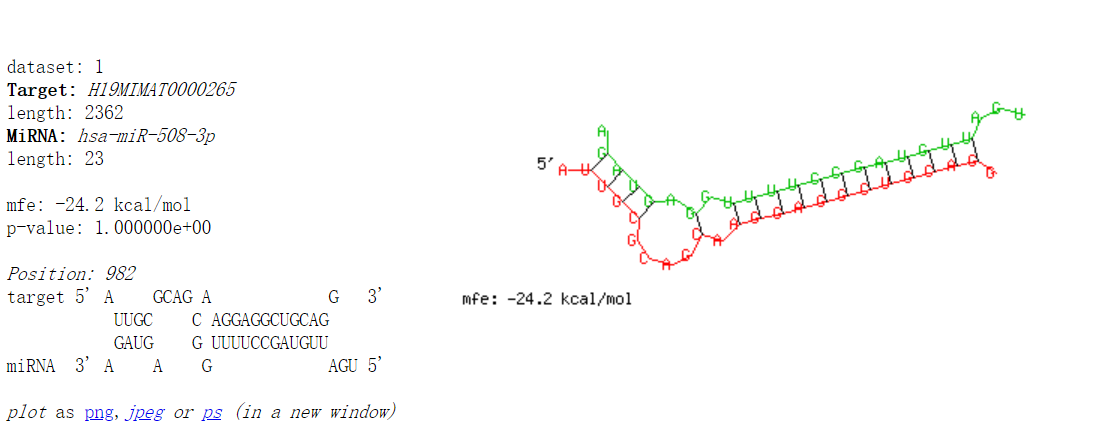


**>hsa-miR-146a-5p MIMAT0000449**

UGAGAACUGAAUUCCAUGGGUU


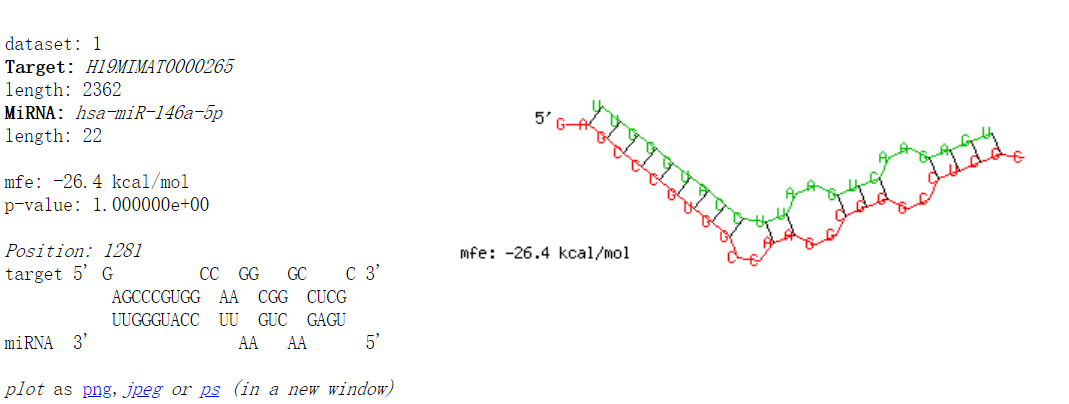


**>hsa-miR-218-5p MIMAT0000275**

UUGUGCUUGAUCUAACCAUGU


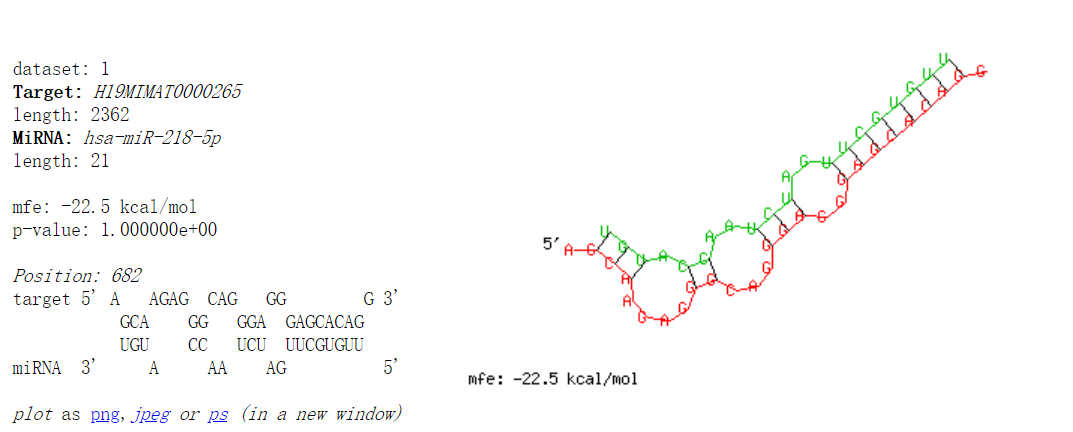


**>hsa-miR-7-5p MIMAT0000252**

UGGAAGACUAGUGAUUUUGUUGUU


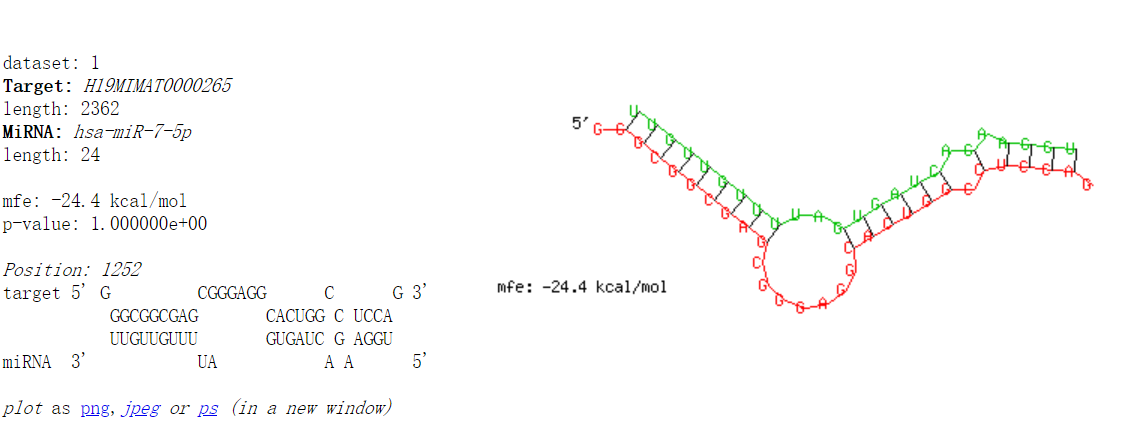


**>hsa-miR-224-5p MIMAT0000281**

UCAAGUCACUAGUGGUUCCGUUUAG


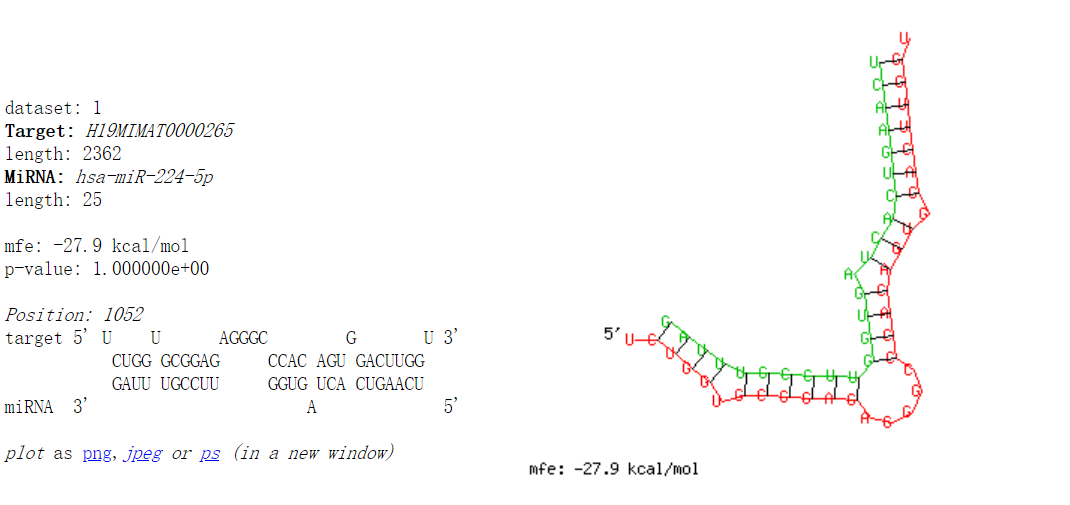


**>hsa-miR-204-5pMIMAT0000265**

**UUCCCUUUGUCAUCCUAUGCCU**


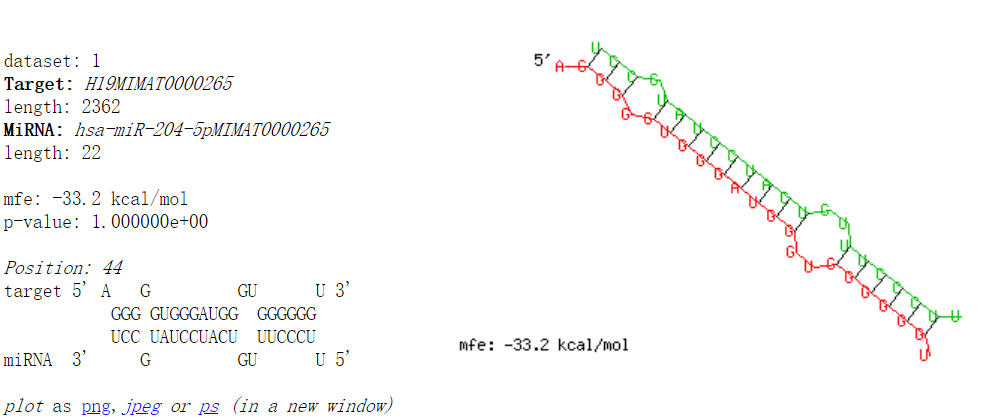

Supplement: Supplementary file 3 [file DataSheet1.docx]
